# BET Bromodomain inhibition promotes De-repression of TXNIP and activation of ASK1-MAPK pathway in acute myeloid leukemia

**DOI:** 10.1186/s12885-018-4661-6

**Published:** 2018-07-11

**Authors:** Yafeng Zhou, Jianbiao Zhou, Xiao Lu, Tuan-Zea Tan, Wee-Joo Chng

**Affiliations:** 1 Cancer Science Institute of Singapore, National University of Singapore, 14 Medical Drive, Centre for Translational Medicine, Singapore, 117599 Republic of Singapore; 2 Department of Medicine, Yong Loo Lin School of Medicine, National University of Singapore, Singapore, 119074 Republic of Singapore; 3 Department of Hematology-Oncology, National University Cancer Institute, NUHS, 1E, Kent Ridge Road, Singapore, 119228 Republic of Singapore

## Abstract

**Background:**

Targeted therapy has always been the focus in developing therapeutic approaches in cancer, especially in the treatment of acute myeloid leukemia (AML). A new small molecular inhibitor, JQ1, targeting BRD4, which recognizes the acetylated lysine residues, has been shown to induce cell cycle arrest in different cancers by inhibiting MYC oncogene. However, the downstream signaling of MYC inhibition induced by BET inhibitor is not well understood.

**Methods:**

In this study, we explored the more mechanisms of JQ1-induced cell death in acute myeloid lukemia and downstream signaling of JQ1.

**Results:**

We found that JQ1 is able to reactivate the tumor suppressor gene, TXNIP, and induces apoptosis through the ASK1-MAPK pathway. Further studies confirmed that MYC could repress the expression of TXNIP through the miR-17-92 cluster.

**Conclusions:**

These findings provide novel insight on how BET inhibitor can induce apoptosis in AML, and further support the development of BET inhibitors as a promising therapeutic strategy against AML.

**Electronic supplementary material:**

The online version of this article (10.1186/s12885-018-4661-6) contains supplementary material, which is available to authorized users.

## Background

Despite the rapid development of targeted therapy in the treatment of different types of cancers, combination chemotherapy remains as the first line therapy in AML. As such, new targeted therapies with fewer side effects are highly desired.

The inhibition of MYC has been shown to be effective by in vitro studies in MYC-driven cancers such as Burkitt lymphoma. Although MYC translocations or mutations are not common in AML, the activation of MYC by multiple tumor-driven genetic aberrations has been recognized as a major factor of leukemogenesis, providing the rationale to target MYC in AML [1].

Although various approaches have been proposed to inhibit MYC, none showed significant clinical benefits. In 2011, Bradner and colleagues developed a small molecular inhibitor named JQ1 which inhibits the bromodomain [2, 3]. JQ1 has been shown to suppress the expression of MYC by inhibiting the chromatin binding subunit of BRD4, causing dissociation of BRD4 from the MYC promoter. It has been shown that JQ1 inhibits proliferation and induces cell cycle arrest in various cancers [4–6].

The mechanism by which JQ1 suppresses the expression of MYC by inhibiting BRD4 has been extensively studied. The disruption of super-enhancer could explain the specific effect of BRD4 inhibition [7]. Many downstream targets of JQ1, such as IL-7R, have been identified in different types of human cancers.

Besides regulating cell cycle, MYC also plays an important role in cell survival and cell fate decision [8]. Hence, it is interesting to examine whether JQ1 is able to cause cell death directly in AML cells. In AML, JQ1 could induce cell death in both cell lines and patient samples [9]. Besides targeting fast dividing cancer cells, JQ1 may also be useful in mitigating the relapse of leukemia through inhibiting the quiescent leukemia stem cells, which are essential contributors of treatment failure and relapse [10].

However, only a few researchers have reported that JQ1 could kill cancer cells besides inducing cell cycle arrest [5, 11, 12]. The detailed mechanisms of how JQ1 induces cell death, particularly in AML, have not been fully uncovered.

The thioredoxin-interacting protein (TXNIP) is a negative regulator of thioredoxin activity. By binding to the catalytic active center of reduced thioredoxin (TRX), TXNIP inhibits its disulfide reductase function, interrupting the antioxidant system, and finally leading to the disruption of redox homeostasis [13].

It has been shown that through its interaction with TRX, TXNIP is involved in the regulation of glucose metabolism, inflammation, and programmed cell death [14–16]. Depleted or repressed TXNIP expression has been reported in breast cancer, non-small cell lung carcinoma, gastric cancer, and colon cancer, and other cancers [17]. Our group has also reported that overexpression of TXNIP is able to induce cell death in AML cells [18].

The current study focused on investigating the mechanism of JQ1-induced cell death and identifying the underlying specific pathway. We demonstrated that JQ1 up-regulates TXNIP expression, followed by activation of ASK1-MAPK pathway, resulting in cell death through intrinsic apoptosis pathway. Furthermore, our data show that TXNIP expression is controlled by MYC through the miR-17-92 cluster. These results not only elucidate the novel mechanism of JQ1-induced apoptosis in AML cells, but also pinpoint the important role of TXNIP in the treatment of AML.

## Methods

### Cell culture

AML cell line Kasumi-1 is kindly provided by Dr. Motomi Osato (CSI, Singapore). All other AML cell lines used in this article, including OCI-AML2 (#ACC99), OCI-AML3 (#ACC582), MOLM-14 (#ACC-777), KG1 (#CCL-246), KG1a (#CCL-246.1), Kasumi-1 (#CRL-2724) and MV4–11 (#CRL-9591), were purchased either from ATCC or DSMZ.

AML cell lines OCI-AML2 and OCI-AML3 were maintained in Minimum Essential Medium α (MEM α) with 20% FBS, 100 U/mL penicillin and 100 μg/mL streptomycin antibiotics. All other AML cell lines were maintained in Roswell Park Memorial Institute (RPMI) -1640 (Invitrogen, Carlsbad, CA) with 10% fetal bovine serum (FBS, JRH Bioscience Inc., Lenexa, KS), 100 U/mL penicillin and 100 μg/mL streptomycin.

### Chemical reagents

JQ1 was kindly provided by Dr. Bradner from Dana-Farber Cancer Institute. MAPK inhibitor SB203580 was purchased from Sigma-Aldrich (s8307). Both of the drug powders were dissolved in dimethyl sulfoxide (DMSO) at 10 mM as stock. CM-H2DCFDA was purchased from Invitrogen (C6827). N-acetyl-l-cysteine (NAC) was obtained from Sigma-Aldrich (A7250). Antibodies against EZH2 (#3147), cleaved PARP (#9541), Caspase-3 (#9661), Caspase-9 (#9501), phosphor-p38 (#9211), p38 (#9212) were purchased from Cell Signaling Technology, antibodies against MYC (sc-40), β-actin (sc-47,778) and secondary antibodies against rabbit (sc-2030) and mouse (sc-2005) were purchased from Santa Cruz Biotechnology, and TXNIP antibody (K0205–3) was purchased from MBL international.

### Drug treatment

Leukemia cells in log phase were washed with 1 × phosphate buffered saline (PBS) for three times and resuspended in appropriate culture media before JQ1 was added. Cells were harvested after 48 h for apoptosis assay measured by Annexin V/PI flow cytometry. In rescue experiments, cells were pretreated with MAPK inhibitor SB203580 or DMSO for 1 h before JQ1 treatment.

### Cell viability assay

The viability of cells under JQ1 treatment was measured using CellTiter-Glo Luminescent Cell Viability Assay Kit (Promega). Cells were seeded in 96- well plate in 2000 cells/well in 100 μl volume. After JQ1 treatment, 50 μL of CellTiter-Glo reagent was added into each well and mixed for 2 mins on an orbital shaker to induce cell lysis. The plate was incubated at room temperature for 10 mins to stabilize luminescent signal. The whole cell lysate was transferred into 96-well white plate prior to measurement with GloMax®-Multi+ Microplate Multimode Reader (Promega).

### Apoptosis assay

Flow cytometry assay was employed to examine the apoptosis of cell lines. For 1 × 10^6^ cells, 1 μl propidium iodide (PI) and 1.5 μl FITC-Annexin V were added and cells were incubated in dark at room temperature for 30 mins. After staining, the FITC and PI fluorescence signals were captured using a BD LSRII flow cytometer (BD Bioscience) and results were analyzed using FlowJo v10.0.8 and Prism GraphPad 5 software.

### Measurement of ROS production

CM-H2DCFDA was used as a cell-permeate indicator for intracellular ROS measurement. AML cells were treated with DMSO, JQ1 or antioxidant N-acetyl-l-cysteine (NAC, 20 mM) for 1 h, followed by JQ1 for additional 48 h. Cells were harvested and resuspended in 1 × PBS containing 10 μM CM-H2DCFDA at 37 °C for 30 min. Cells were washed with 1 × PBS, then analyzed using FITC channel in a BD LSRII flow cytometer.

### Real time quantitative PCR for mRNA and miRNA

For quantification of mRNA and miRNA levels, real time qPCR was performed as described before [19]. GAPDH and human U6 were used as the internal controls for mRNA and miRNA, respectively. The primers sequences were: MYC-For: 5’-AATGAAAAGGCCCCCAAGGTAGTTATCC-3′, MYC-Rev: 5’-GTCGTTTCCGCAACAAGTCCTCTTC-3′, TXNIP-For: TCATGGTGATGTTCAAGAAGATC, TXNIP-Rev: ACTTCACACCTCCACTATC, miR-17-92-For: CTGTCGCCCAATCAAACTG, miR-17-92-Rev: GTCACAATCCCCACCAAAC, GAPDH-For: GTATTGGGCGCCTGGTCAC, GAPDH-Rev: CTCCTGGAAGATGGTGATGG.

### Western blot

Protein extraction and Western blot were performed as described before [20]. Densitometric analysis was performed using ImageJ v1.49 (National Institutes of Health, US).

### Caspase activity assay

The activities of caspase-3 and caspase-7 were measured using Caspase-Glo 3/7 assay (Promega). Cells were seeded in 96- well plate in 2000 cells/well in 100 μl volume. After JQ1 treatment, 50 μL of Caspase-Glo 3/7 reagent was added into each well and mixed for 30 mins on plate shaker at room temperature to induce cell lysis. The lysates were then transferred into white-walled 96-well plate prior to recording luminescence with GloMax®-Multi+ Microplate Multimode Reader (Promega).

### TXNIP gene expression and CHNG_MYC signature

The TXNIP gene expression was extracted from an acute myeloid leukemia (AML) meta-cohort consists of 1149 samples compiled previously [PMID: 25214461]. Briefly, microarray data of human acute myeloid leukemia (AML) on Affymetrix U133A or U133Plus2 platforms were downloaded from Gene Expression Omnibus (GEO). Robust Multichip Average (RMA) normalization was performed on each dataset and subsequently standardized using ComBat [Ref: Johnson] to remove batch effect. The 7-gene MYC signature was estimated by taking the average expression of all the seven genes from the AML meta-cohort [PMID: 21468039].

## Results

### JQ1 inhibits growth of AML cell lines

**Fig. 1:**
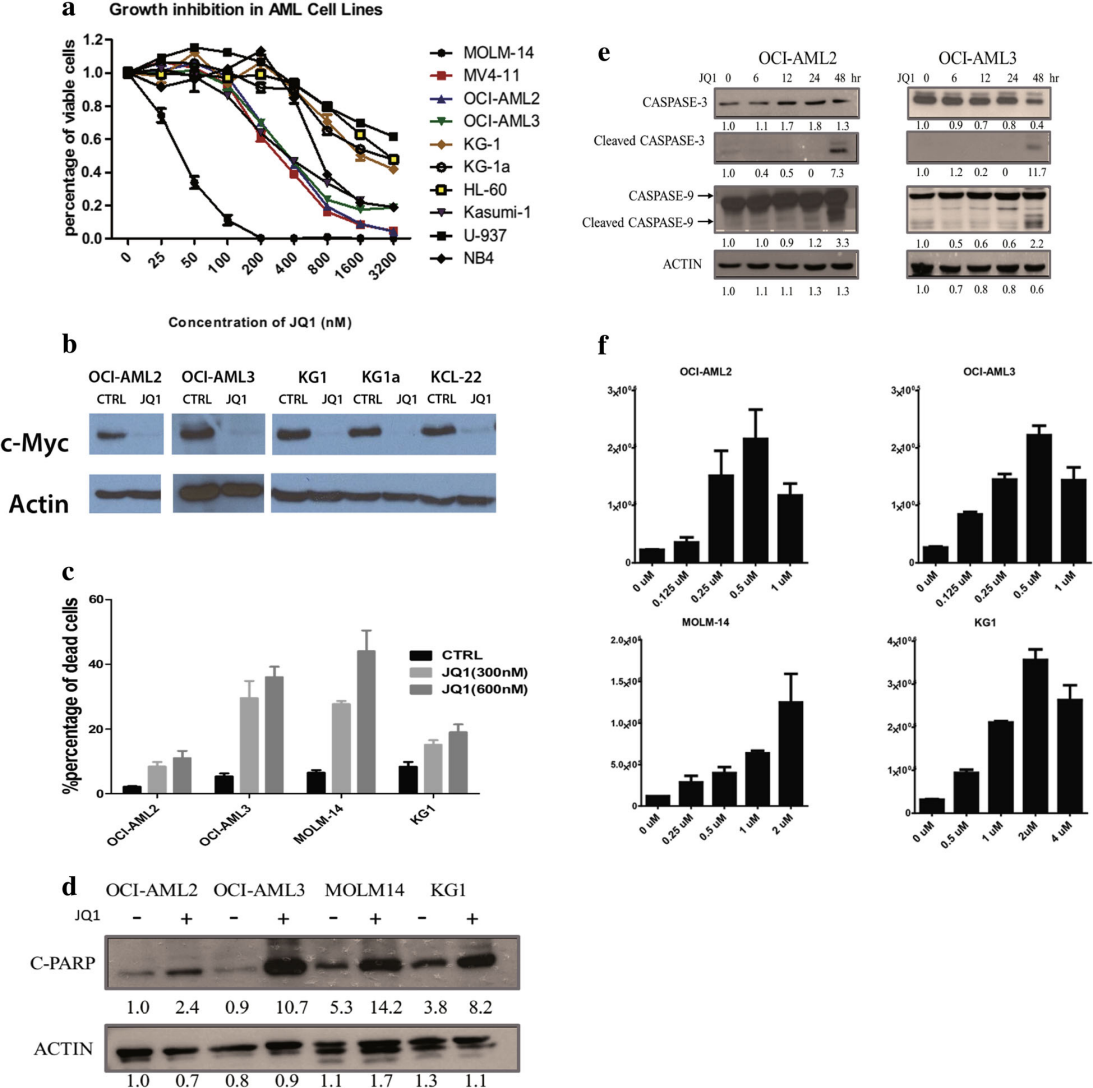
JQ1 inhibits growth and induces apoptosis in AML cell lines. **a** AML cell lines were treated with JQ1 at gradient concentration for 48 h and the viabilities were measured using CellTiter-Glo. The growth rates were normalized to cells without treatment. **b** Change of c-Myc protein level after JQ1 treatment for 48 h. **c** AML cell lines were treated with JQ1 at 300 nM or 600 nM for 48 h before they were stained with PI and Annexin V and analyzed using flow cytometry. Percentages of apoptotic cells at early and late stage (i.e. Annexin V positive) were shown. **d** C-PARP protein level significantly increased after JQ1 treatment for 24 h. **e** Activation of intrinsic apoptosis pathway. OCI-AML-2 and OCI-AML-3 Cells were treated with JQ1 at respective IC_50_ and harvested at different time points before immunoblotting. **f** Caspase activity assay of dose-dependent JQ1-treated AML cell lines. For each concentration, triplicate samples were prepared. The relative activities were normalized to culture medium

To investigate the effect of JQ1 in AML, we first verified whether JQ1 impairs the growth of AML cell lines. Cells were treated with JQ1 at different concentrations for 48 h, followed by CellTiter-Glo assay. These results confirmed that JQ1 treatment dose-dependently inhibits the growth of AML cell lines (Fig. 1a). Furthermore, the IC_50_ of JQ1 in most cell lines were less than 1 μM, which suggest strong anti-leukemic effect of JQ1 in AML cells (Table 1).

**Table 1:** IC-50 and genetic rearrangements for AML cell lines tested

Genetic rearrangement	Cell lines	IC50 (nM)
t (4;11) (q21; q23)	MOLM-14	45
t (8;21) (q22; q22)	Kasumi-1	222
t (4;11) (q21; q23)	MV4-11	245
hypodiploid	OCI-AML3	260
hypodiploid	OCI-AML2	309
t (10;11) (p12; q14)	U937	569
hypodiploid	KG1a	628
t (15;17) (q22; q12)	NB4	710
hypodiploid	KG1	775
hypodiploid	HL-60	1222

In the study of another BET inhibitor, I-BET151, the anti-leukemic effect is found to be associated with the MLL translocation status. Based on our results, the sensitivity to JQ1 is unrelated to the chromosome status. Although cells with MLL translocation (e.g. MOLM-14 and MV4–11) showed strong response to JQ1 treatment, cells without MLL translocation (e.g. Kasumi-1 and OCI-AML3) are also sensitive to JQ1. Therefore, it is important to further study the mechanisms of JQ1 in AML.

As MYC has been reported to be an important target of JQ1, we checked for a change in MYC expression upon JQ1 treatment. Consistent with previous reports, Western blot analysis showed high MYC expression in all AML cell lines (Fig. 1b), which was largely depleted after 24 h of JQ1 treatment.

### JQ1 induces apoptosis in AML cells

In AML, it has been shown that JQ1 induces cell death, but little is known about its mechanism [9, 21] Therefore, we examined whether JQ1 induces cell death in AML cells and investigated the underlying mechanism.Four AML cell lines, OCI-AML2, OCI-AML3, MOLM-14, and KG-1, were treated with JQ1 at 300 nM and 600 nM for 48 h prior to analysis with flow cytometry assay. Based on FACS analysis, JQ1 induced strong apoptosis in all AML cells in a dose-dependent manner (Fig. 1c). This was further confirmed by Western blot analysis of cleaved PARP, which is a hallmark of apoptosis (Fig. 1d). We also investigated whether the apoptosis is mediated via the extrinsic or intrinsic pathway. After 48 h of JQ1 treatment, both OCI-AML2 and OCI-AML3 showed significant increase in cleaved caspase-9 and -3 proteins, demonstrating the activation of the intrinsic apoptosis pathway (Fig. 1e). To validate the activation of the apoptotic pathway, caspase activities were also examined using Caspase-Glo 3/7 assay. The activities of Caspase-3 and Caspase-7 increased in a dose-dependent manner after JQ1 treatment for 48 h in AML cells (Fig. 1f). Taken together, these results demonstrate that JQ1 leads to the activation of the intrinsic apoptosis pathway in AML cells.

### TXNIP is a key mediator in JQ1-induced apoptosis

To investigate the mechanism of JQ1-induced apoptosis in AML, we analyzed the published gene expression profile data of JQ1-treated cells [2, 4, 22]. We identified a set of genes with expressions that were consistently changed upon JQ1 treatment (Additional file 1: Figure S1A and B). While MYC, the known target of JQ1, is amongst the down-regulated genes, HEXIM1, a negative regulator of P-TEFb complex, is one of the up-regulated genes. HEXIM1 has been reported to be upregulated upon JQ1 treatment [23]. These known genes suggest that other genes may be relevant and are worthy of further study.

We have previously reported that TXNIP, one of the up-regulated genes, to be an important mediator of cell death in AML caused by an EZH2 inhibitor, 3-Deazaneplanocin A [18].

**Fig. 2:**
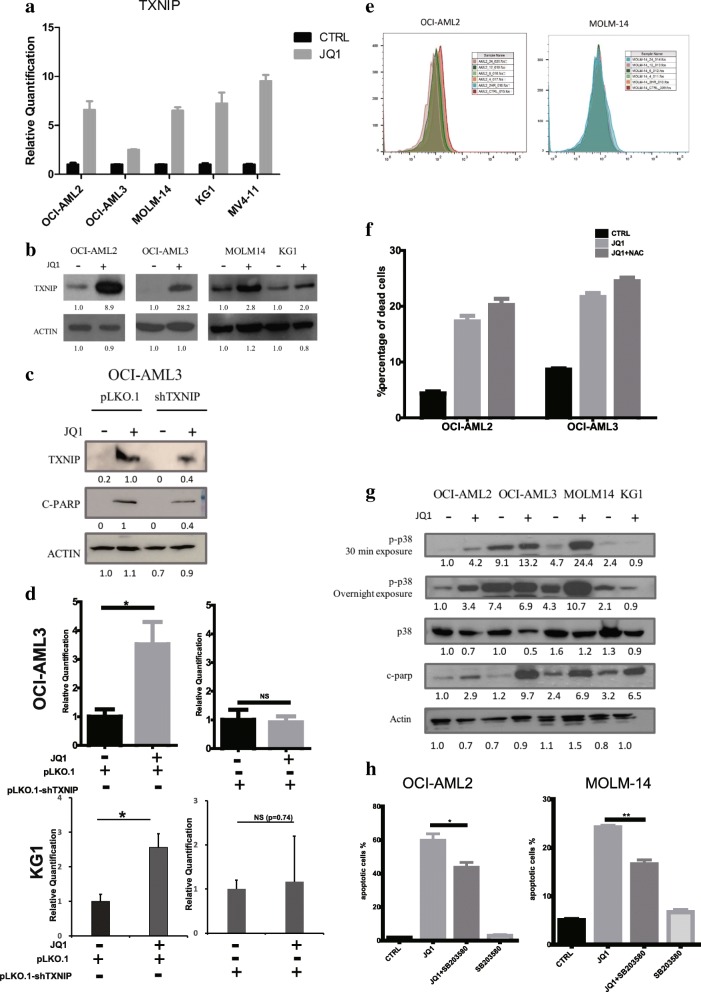
TXNIP partially mediates the JQ1-induced apoptosis through activating the p38 MAPK pathway instead of increasing ROS. TXNIP (**a**) mRNA and (**b**) protein level after JQ1 treatment. Cells were treated with JQ1 at respective IC_50_ for 24 h before being harvested and lysed. For real time qPCR results, means and standard errors were shown. **c** OCI-AML-3 cells were transfected with either pLKO.1 or pLKO.1-shTXNIP vectors and treated with JQ1 or DMSO for 24 h. **d** Relative quantification of apoptotic cells percentage using flow cytometry. Triplicates were performed and standard errors were shown. Percentages of apoptotic cells were normalized to DMSO-treated control. **e** Flow cytometry results showing the ROS level change in OCI-AML2 and MOLM-14 cells. Cells were treated with JQ1 at respective IC_50_ and harvested at different time points. **f** Percentages of apoptotic cells measured by flow cytometry. Cells were pre-treated with NAC for 1 h before adding JQ1. **g** AML cells were treated with JQ1 for 24 h and subjected to immunoblot analysis with primary antibodies indicated. **h** p38 MAPK inhibitor rescued cells from JQ1-induced apoptosis. Percentages of apoptotic cells were shown (*: *p* < 0.05, **: *p* < 0.01)

We first validated the expression of TXNIP before and after JQ1 treatment. Both the mRNA and protein levels of TXNIP increased upon JQ1 treatment across multiple cell lines (Fig. 2a, b). To study whether TXNIP plays a role in JQ1-induced apoptosis, OCI-AML3 cells were transfected with scramble shRNA or shRNAs targeting TXNIP, followed by JQ1 treatment. The cells were examined using Western blot and flow cytometry for apoptosis. Knocking down TXNIP can rescue the apoptosis caused by JQ1 in AML cells, with a significant lower level of cleaved PARP and a dramatic reduction on apoptotic cell percentages (Fig. 2c, d). This suggests that cell death induced by JQ1 is largely mediated by the induction of TXNIP expression. As TXNIP negatively regulates thioredoxin and perturbs the redox control system, we examined if this pathway is involved in the JQ1-mediated apoptosis of AML cells by quantifying the production of reactive oxygen species (ROS) following JQ1 treatment.

OCI-AML2 and MOLM-14 cells treated with JQ1 at the respective IC_50_ were collected at different time points and stained with H2-DCFDA followed by flow cytometry analysis (Fig. 2e and Additional file 1: Figure S1C). After JQ1 treatment, ROS level decreased in OCI-AML2, and slightly increased in OCI-AML3, which indicates that ROS may not play a role in TXNIP-mediated apoptosis. To confirm, a ROS scavenger, N-acetyl-L-cysteine (NAC), was employed to rescue the JQ1-induced apoptosis. As expected, NAC failed to rescue the apoptosis induced by JQ1, with a similar percentage of apoptotic cells in comparison with cells pre-treated with DMSO (Fig. 2f). Taken together, these findings suggest that elevated ROS level is not involved in JQ1-induced cell death and TXNIP might function through other mechanisms.

TRX2 has also been shown to be a target of TXNIP and its inhibition causes activation of mitochondrial apoptosis through the MAPK pathway. The phospho-p38 levels were determined using Western blot. The results show that upon JQ1 treatment, phospho-p38 level in most cell lines increased significantly while the p38 levels decreased slightly, which was also consistent with the JQ1-induced apoptosis shown by cleaved-PARP (Fig. 2g). To validate the role of phospho-p38 in JQ1-induced apoptosis, a phospho-p38 inhibitor, SB203580 was used to inhibit the function of phospho-p38. Cells co-treated with JQ1 and SB203580 showed a significant reduction in the percentage of apoptotic cells compared to those treated with JQ1 alone, suggesting an important role of phospho-p38 in mediating JQ1-induced apoptosis (Fig. 2h). In summary, JQ1 elevates TXNIP proteins and induces apoptosis mainly through the ASK-MAPK pathway.

### TXNIP is a downstream target of MYC

As an important regulator of the TRX system, various factors have been proposed to regulate TXNIP expression, including glucose flux, oxidative stress, and ceramide treatment [16, 24, 25]. However, the exact mechanism of TXNIP regulation in AML remains elusive.

MYC has been reported as an important downstream target of BRD4 and its level decreases after JQ1 treatment. Therefore, we investigated the role of MYC in the regulation of TXNIP expression.

**Fig. 3:**
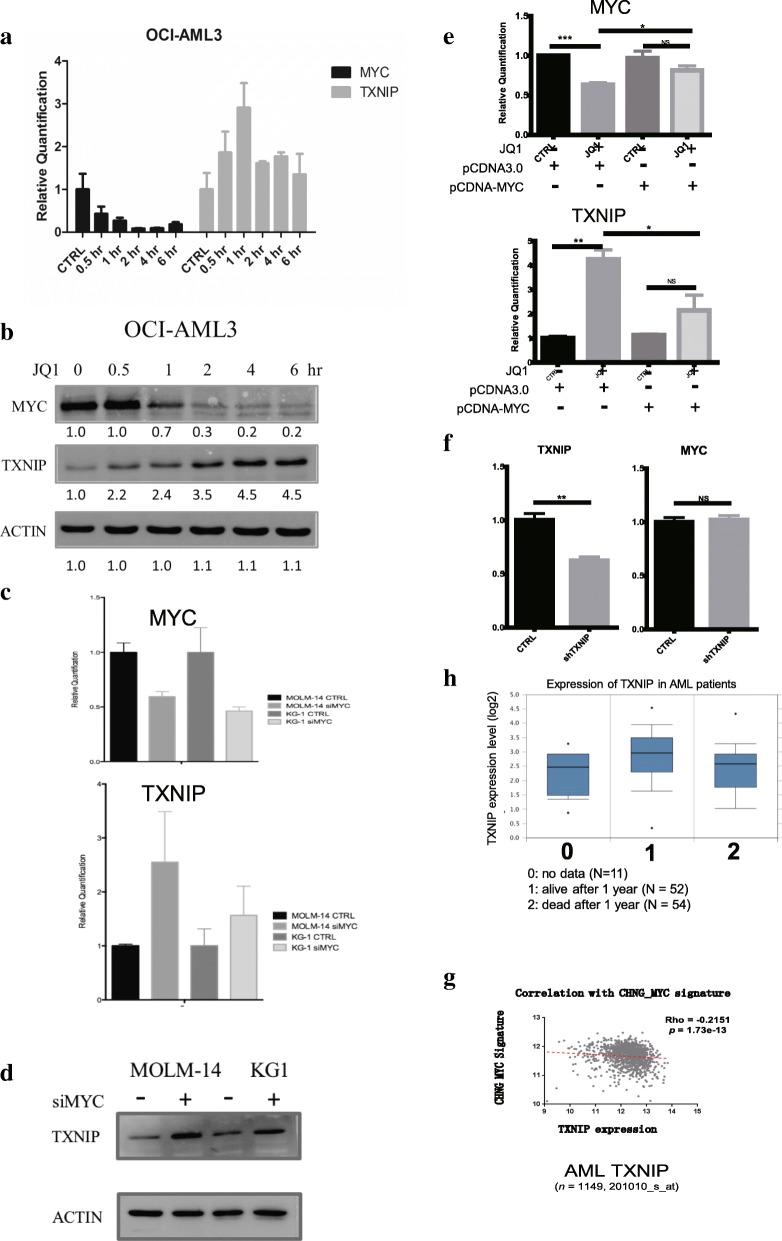
MYC regulates TXNIP levels upon JQ1 treatment. The (**a**) mRNA and (**b**) protein level of MYC and TXNIP after JQ1 treatment. **c** The mRNA levels of MYC and TNXIP after knocking-down MYC in MOLM-14 and KG1 cells. Cells were transfected with siRNAs targeting MYC using NEON transfection system analyzed with qRT-PCR. **d** The protein levels of TXNIP after knocking-down MYC in MOLM-14 and KG1 cells. **e** KG1 cells were transfected with either pCDNA3.0 or pCDNA3-MYC vectors and treated with DMSO or JQ1 for 24 h. **f** The expression levels of TXNIP and MYC signature were analyzed using spearman’s correlation test. **g** T Dot plot of MYC signature (y-axis) and TXNIP gene expression (x-axis) in AML meta-cohort (*n* = 1149). Linear regression fit is shown as red dotted line. *P*-value is computed by Spearman correlation coefficient test. **h** TXNIP expression levels in AML patients alive or dead 1 year after diagnosis (0: no data available, *N* = 11, median = 2.47, VAR = 0.58; 1:alive after 1 year, *N* = 52, median = 2.96, VAR = 0.92; 2: dead after 1 year, *N* = 54, median = 2.58, VAR = 0.88. *p* = 0.0093 (1 v.s. 2, non-parameter test)

The changes in mRNA and protein levels of MYC and TXNIP were recorded at various time points up to 6 h after JQ1 treatment in OCI-AML3 cells. There was a prompt reduction of MYC expression in parallel to an increase in TXNIP expression (Fig. 3a, b).

To confirm the relation between MYC and TXNIP, MYC expression was knocked down in MOLM-14 and KG1 using siRNAs targeting MYC. The mRNA levels of MYC and TXNIP were examined using qRT-PCR. After knocking down MYC, the mRNA level of TXNIP increased significantly in both cell lines, which indicates that MYC might be the repressor of TXNIP in AML (Fig. 3c). Western blot analysis substantiated these observations (Fig. 3d). In cells with ectopic expression of MYC, JQ1 treatment no longer induce any significant change in TXNIP expression (Fig. 3e). Furthermore, KG1 cells transfected with shRNAs targeting TXNIP showed no changes in MYC level, indicating that MYC is the upstream regulator of TXNIP (Fig. 3f).

To prove the relationship between MYC and TXNIP in AML patients, the correlation between MYC activation and TXNIP expression was analyzed. We have previously generated an MYC signature consisting of a panel of genes activated by MYC [26]. Using this signature, we have demonstrated an inverse correlation between the MYC activation signature and the expression level of TXNIP in a cohort of more than 1000 AML patients (Fig. 3g). This suggests that our observation of relationship between MYC and TXNIP in cell lines is also relevant in clinical samples. The connection between TXNIP expression and clinical outcome of AML patients was also studied. Using a cohort data from Oncomine [27], we found that patients who were alive 3 years after diagnosis have much higher TXNIP level comparing to those who died (Fig. 3h). Taken together, these results suggest that TXNIP is a novel downstream target of MYC.

### MYC regulates TXNIP through miRNAs

We have identified the important regulatory role of MYC in TXNIP expression. However, the mechanism by which MYC represses the expression of TXNIP is still unknown and therefore needs to be investigated. As miR-17-92 has been reported to be an important target of MYC, we wanted to explore whether it also participates in the regulation of TXNIP. Therefore, we first analyzed the 3’UTR of TXNIP for miRNA binding sites using TargetScan. Three of the six miRNAs from the miR-17-92 cluster (miR-17, miR-18a, miR-19a, miR-19b-1, miR-20a, miR-92a-1), miR-17, miR-18a and miR-20a, were predicted to have highly conserved binding sites on the 3’UTR of TXNIP (Additional file 1: Figure S1D).

**Fig. 4:**
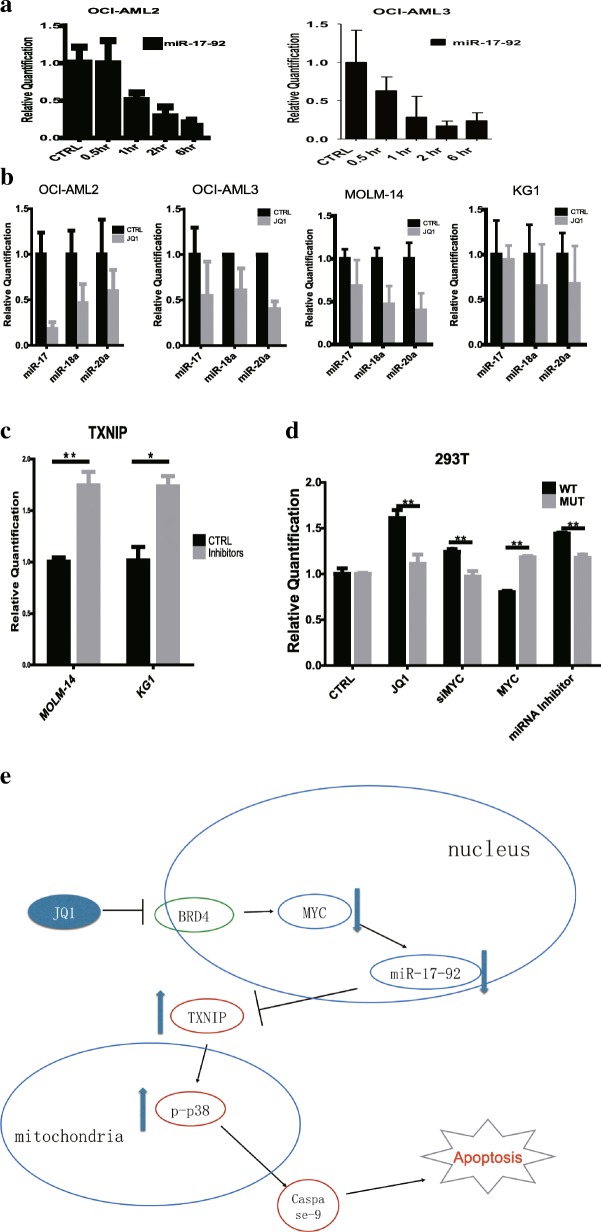
miR17–92 cluster mediates the regulation between MYC and TXNIP. **a** JQ1 treatment repressed the expression of miR-17-92 rapidly. **b** The expressions of mature miRNAs from the miR-17-92 cluster were inhibited by JQ1 treatment. **c** MOLM-14 and KG1 cells were transfected with miRNA inhibitors, cultured for 24 h, and the expression level of TXNIP was examined. **d** 293 T cells were transfected with luciferase vectors carrying wild type (WT) of mutant (MUT) 3’UTR of TXNIP before treatment with JQ1 (2 μM), transfected with MYC siRNAs, MYC expressing vector, or miRNA inhibitors. Cells were cultured for 24 h under different treatments before being harvested for luciferase activity assay. The final luciferase activities were normalized to total protein concentration and measured with Bradford assay. **e** Proposed model of MYC-TXNIP regulatory cascade

To confirm the relationship between miR-17-92 and TXNIP, two cell lines, OCI-AML2 and OCI-AML3, were treated with JQ1 for 6 h and the levels of miR-17-92 transcript and mature miRNAs at different time points were checked using real time PCR. Both the level of miR-17-92 transcript and mature miRNAs decreased significantly after JQ1 treatment (Fig. 4a, b).

To find out whether these miRNAs regulate the expression of TXNIP, miRNA inhibitors specific to this cluster were transfected into MOLM-14 and KG1 cells. Figure 4c shows that the inhibition of miR-17, miR-18 and miR-20 significantly increased the mRNA level of TXNIP, which indicates the regulatory role of these miRNAs in controlling TXNIP expression.

To validate the direct targeting of TXNIP by these miRNAs, HEK-293 T cells were transfected with plasmids carrying either wild type or mutant TXNIP 3’UTR (Additional file 1: Figure S1E), followed by different treatments. Following JQ1 treatment or MYC knock-down, there was a significant increase in luciferase activities. The similar observation recorded with miRNA inhibitors suggest that this effect is specific to the inhibition of miRNA following JQ1 treatment. On the other hand, overexpression of MYC led to a decrease in the luciferase activities. Meanwhile, in cells transfected with mutant TXNIP 3’UTR, the effects of different treatments were significantly abolished (Fig. 4d). Taken together, these results provide further evidence supporting our hypothesis that MYC controls expression of TXNIP through these miRNAs (Fig. 4e).

## Discussion

JQ1 has been shown to inhibit growth through suppression of MYC expression, resulting in disruption of MYC regulatory network. Many downstream targets of MYC, such as p21, CDK4, and CCDC86, have been demonstrated to play important roles in JQ1-induced cell cycle arrest [16, 28, 29]. It has also been reported that JQ1 eliminates leukemic stem and progenitor cells in AML, showing its anti-survival effect [9]. Other available BET/BRD4 inhibitor, such as AZD5153, were also shown strong anti-tumor effect [30].

In this study, we characterized the pro-apoptotic effect of the small molecular drug, JQ1, in AML cells. Besides inducing cell cycle arrest, JQ1 could also activate the intrinsic apoptosis pathway to at least partially through the up-regulation of TXNIP. We also demonstrated that activation of the p38 MAPK pathway plays a role in JQ1-induced apoptosis through TXNIP. We therefore proposed a possible explanation of JQ1-induced apoptosis through the down-regulation of MYC, up-regulation of TXNIP, and the activation of p38 MAPK, which may provide further understanding of this process.

While TXNIP has been known for its role in the regulation of glucose metabolism and cellular redox status, its role in tumor repression was relatively less studied. The expression level of TXNIP is decreased in many types of cancer compared to the normal tissues. We have previously reported that mRNA and protein of TXNIP to be lower in either leukemia cell lines or primary bone marrow cells from patients with AML. Moreover, overexpression of TXNIP induces apoptosis in AML cells. In this study, we demonstrated that the up-regulation of TXNIP is, at least partially, responsible for the JQ1-induced cell death, which further supports the role of TXNIP as a tumor suppressor. Interestingly, it has been reported that TXNIP level is associated with HSC population and the differentiation of normal hematopoietic cells [31–33].

Despite being a well-studied gene regulator, the association between MYC and TXNIP has not been much reported. We revealed that the epigenetic regulation of TXNIP by MYC through the miR-17-92 cluster. The miR-17-92 cluster has also been shown to mediate MYC’s role in maintaining the survival, proliferation, and neoplastic state in hematopoietic malignancies. Our findings further support these observations by adding TXNIP and apoptosis into the complex MYC-regulatory network. This is also consistent with another study in triple-negative breast cancer that TXNIP level is inversely correlated to MYC level, whereby MYC could repress expression of TXNIP [34].

## Conclusions

Taken together, our study provide more evidences for the cell death function of JQ1 and reveals a potential mechanism of JQ1-induced apoptosis in AML through the up-regulation of a tumor suppressor TXNIP. This study also might be helpful to further understand the action of BET inhibitors.

## Additional file

Additional file 1: Supplementary information. (DOCX 197 kb)

## Abbreviations

AML: Acute myeloid leukemia
BET: Bromodomain and extraterminal domain
BRD4: Bromodomain containing protein 4
MAPK: Mitogen-activated protein kinases
MYC: V-mycavian myelocytomatosis viral oncogene homolog
ROS: Reactive oxygen species
TXNIP: Thioredoxin-interacting protein
