# Supplementary material for: BET Bromodomain inhibition promotes De-repression of TXNIP and activation of ASK1-MAPK pathway in acute myeloid leukemia
Source: BMC Cancer. 2018 Jul 11;18:731. doi: 10.1186/s12885-018-4661-6 (PMC6042241; doi:10.1186/s12885-018-4661-6)
Supplement: Supplementary file 1 — Supplementary information. (DOCX 197 kb) [file 12885_2018_4661_MOESM1_ESM.docx]

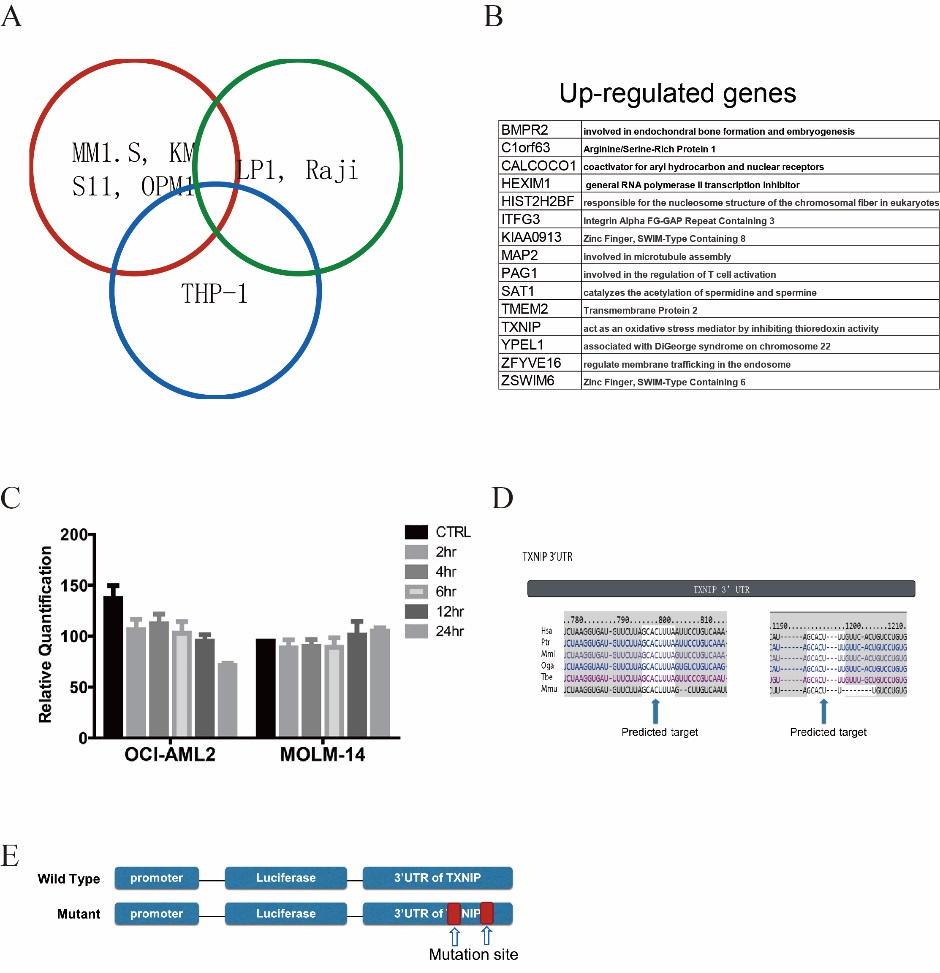


**Supplementary Figure 1.** (A) Three mRNA microarray studies using six different cell lines. (B) Genes commonly upregulated in all data sets. (C) The relative ROS levels in OCI-AML2 and MOLM-14 cells after JQ1 treatment. (D) 3’UTR of TXNIP were predicted to contain two binding sites of miRNAs from miR-17-92 cluster by TargetScan 7.0. (E) 293T cells were transfected with luciferase vectors carrying wild type (WT) of mutant (MUT).

**Table 1**

| **Genetic rearrangement** | **Cell lines** | **IC50 (nM)** |
| --- | --- | --- |
| t(4;11)(q21;q23) | MOLM-14 | 45 |
| t(8;21)(q22;q22) | Kasumi-1 | 222 |
| t(4;11)(q21;q23) | MV4-11 | 245 |
| hypodiploid | OCI-AML3 | 260 |
| hypodiploid | OCI-AML2 | 309 |
| t(10;11)(p12;q14) | U937 | 569 |
| hypodiploid | KG1a | 628 |
| t(15;17)(q22;q12) | NB4 | 710 |
| hypodiploid | KG1 | 775 |
| hypodiploid | HL-60 | 1222 |
